# The Critical Role of Long Noncoding RNA in Osteogenic Differentiation of Human Bone Marrow Mesenchymal Stem Cells

**DOI:** 10.1155/2017/5045827

**Published:** 2017-04-27

**Authors:** Xiaoling Qiu, Bo Jia, Xiang Sun, Weitao Hu, Hongxing Chu, Shuaimei Xu, JianJiang Zhao

**Affiliations:** ^1^Department of Endodontics, Stomatological Hospital of Southern Medical University and Guangdong Provincial Stomatological Hospital, Guangzhou 510280, China; ^2^Department of Oral Surgery, Stomatological Hospital of Southern Medical University and Guangdong Provincial Stomatological Hospital, Guangzhou 510280, China

## Abstract

*Objective*. Long noncoding RNAs (lncRNAs) have been demonstrated to regulate many biological processes including differentiation. However, their role in osteogenic differentiation was poorly known.* Materials and Methods*. In this study, we first globally profiled the differentially expressed lncRNAs and mRNAs during osteogenic differentiation of human bone marrow mesenchymal stem cells (hBMMSCs). Bioinformatics analysis was performed to further analyze these significantly changed molecules. Then the role of lncRNA ENST00000502125.2 in the osteogenic differentiation was determined.* Results*. A number of lncRNAs and mRNAs were significantly differentially expressed during hBMMSC osteogenic differentiation. Among them, 433 lncRNAs and 956 mRNAs were continuously upregulated, while 232 lncRNAs and 229 mRNAs were continuously downregulated. Gene Ontology and KEGG (Kyoto Encyclopedia of Genes and Genomes) analysis showed that carbohydrate derivative binding and complement and coagulation cascades were most correlated molecular function and pathway, respectively. Downregulation of lncRNA ENST00000502125.2 promoted the osteogenic differentiation of hBMMSCs, and opposite results were found when lncRNA ENST00000502125.2 was upregulated.* Conclusions*. lncRNAs play a critical role in the osteogenic differentiation of hBMMSCs and targeting lncRNA ENST00000502125.2 might be a promising strategy to promote osteogenic differentiation.

## 1. Introduction

In the craniomaxillofacial skeleton, bone defect is still a major and challenging health concern. Its size can vary from the very small periodontal defects to the large segmental defects [1]. Efficacious bone tissue engineering requires a combination of three key factors including stem cells, growth factors, and scaffolds [2]. Human mesenchymal stem cells (hMSCs) are adult stem cells that can be isolated from adult tissues such as bone marrow, adipose tissues, and dental pulp [3, 4]. In addition to colony formation and self-renewal capability, hMSCs have the capacity to differentiate into a number of cell types such as osteoblasts, adipocytes, chondrocytes, and neural-like cells [5, 6]. Moreover, the immunosuppressive potential of hMSCs is important for the treatment of graft-versus-host diseases [7] These intrinsic properties of hMSCs make them an ideal cell source for both cellular therapy and regenerative medicine.

Long noncoding RNAs (lncRNAs) are a class of non-protein-coding transcripts with a length of more than 200 nucleotides [8]. Similarly to microRNAs, lncRNAs have been demonstrated to play a central role in regulating many important biological processes such as cell proliferation, differentiation, migration, and development [9, 10]. Mutations and deregulations of lncRNAs link to the initiation and progression of diverse human diseases ranging from cardiovascular diseases to cancer [11]. Various studies reported that lncRNAs are important for the osteogenic differentiation of MSCs. Downregulation of lncRNA MEG3 significantly suppressed the osteogenic potential of MSCs, and opposite findings were detected when lncRNA MEG3 was overexpressed. In addition, its regulatory effects on osteogenic differentiation were achieved by partly activating BMP4 [12]. Similarly, lncRNA MIR31HG inhibition promoted the osteogenic differentiation of human adipose-derived stem cells (hASCs) in either normal or inflammatory environment by regulating NF-*κ*B signalling pathway, indicating lncRNA MIR31HG- NF-*κ*B axis is critical for the osteogenic differentiation of hASCs [13].

However, the function of lncRNAs in the osteogenic differentiation of human bone marrow MSCs (hBMMSCs) remains poorly known. Therefore, the purpose of the current study was global profiling of the changes of mRNA and lncRNA during the osteogenic differentiation of hBMMSCs and to determine the role of lncRNA ENST00000502125.2 in the process of osteogenesis.

## 2. Materials and Methods

### 2.1. Cell Culture

HBMMSCs were purchased from ScienCell Research Laboratories (Cat. number 7500; Carlsbad, CA, USA). The hBMMSCs were maintained with Mesenchymal Stem Cell Medium (ScienCell, Carlsbad, CA, USA) at 37°C in a humidified atmosphere of 95% air and 5% CO2. For the osteogenic differentiation of hBMMSCs, the cells were cultured in osteogenic medium containing 10 mmol/L *β*-glycerol phosphate, 50 *μ*g/mL ascorbic acid, and 10−7 mol/L dexamethasone.

### 2.2. Real-Time PCR

Total RNA was isolated from hBMMSCs using TRIzol (Invitrogen, Carlsbad, CA, USA) according to the manufacturer's instructions. First-strand cDNA was synthesized from 2 *μ*g RNA using the SuperScript®III Reverse Transcriptase (Invitrogen). Then the cDNA levels were amplified and quantified with SYBR Green PCR master mix (Applied Biosystems, Foster City, CA, USA). The PCR reaction was performed using the 7900HT Fast Real-Time PCR System (Applied Biosystems). Gene expression was normalized against glyceraldehyde-3-phosphate dehydrogenase (GAPDH). The primer sequence was summarized in Supplementary Table 1 (in Supplementary Material available online at https://doi.org/10.1155/2017/5045827).

### 2.3. Chip Arrays

Human 4 × 180 K lncRNA arrays contained 37581 lncRNAs and 34235 mRNAs. Total RNA was obtained as described above. Approximately 200 ng of total RNA from each sample was used for the microarray analyses. Briefly, followed by spiking with an RNA Spike-In Kit with one color (Agilent), total RNA was reversely transcribed into cDNA and then converted into Cyanine-3 labeled cRNA. Cyanine-3-labeled cRNA sample was fragmented and then hybridized at 65°C for 17 h using an Agilent Gene Expression Hybridization Kit in hybridization chamber gasket slides (Agilent). After hybridization, the microarrays were washed with an Agilent Wash Buffer kit (Agilent) and scanned with an Agilent microarray scanner. The resulting images were analyzed using Agilent's Feature Extraction software v10.7 and Agilent GeneSpring.

### 2.4. Gene Function Analysis

To investigate the potential functions of these lncRNAs, the predicted target genes were input into the Database for Annotation, Visualization and Integrated Discovery (DAVID; http://david.abcc.ncifcrf.gov/), which utilized Gene Ontology (GO) to identify the molecular function. In addition, KEGG (Kyoto Encyclopedia of Genes and Genomes) database (http://www.genome.ad.jp/kegg/) was employed to analyze the potential functions of these target genes in the pathways. Lower *p* value indicates that the correlation is more significant. The cut-off *p* value is 0.05.

### 2.5. Lentivirus Construction and Transfection

The lncRNA ENST00000502125.2-targeting (5′-GGTTAATCCACAGTTAGGAGCTTCCTGTCAGACTCCTAACTGTGGATTAACCTTTTT-3′)/overexpression (5′-TTCCCCATCCACAGAAATGGT-3′) oligonucleotide sequences were cloned into the lentiviral vectors. The mock control consisted of a scrambled sequence with no homology to any gene in the human genome. Recombinant lentiviral vectors and packaging vectors were then transfected into 293T cells. The supernatant containing lentiviruses was harvested 72 h after transfection. The lentiviruses were then purified by ultracentrifugation, and the titer of lentiviruses was determined. Human BMMSCs were infected with the lentiviruses at a multiplicity of infection of 50.

### 2.6. ALP Staining

About 2 × 10^5^ cells/plate were seeded in six-well plates and subjected to osteogenic differentiation induction when the cell density reached 70–80% confluence. ALP staining was performed with an ALP staining kit (Sigma-Aldrich) at day 7 according to the manufacturer's protocols. Briefly, the induced cells were fixed in 4% formaldehyde and incubated with a substrate buffer at room temperature (RT) for 30 minutes. The reaction was terminated by removing the substrate solution and washing with PBS. The staining results were observed under microscope.

### 2.7. Alizarin Red S Staining

About 2 × 10^5^ cells/plate were seeded in six-well plates and subjected to osteogenic differentiation induction when the cell density reached 70–80% confluence. The induced cells at day 21 were gently washed with PBS twice and then fixed in 4% formaldehyde for 30 min at RT. Then cells were washed with distilled water three times, stained with 2% Alizarin Red S for 30 min at RT, and washed with distilled water to remove the remaining staining. The staining results were observed under microscope.

### 2.8. Statistical Analysis

The data were analyzed and expressed as means ± standard deviation. All the experiments were performed in triplicate and repeated three times. The threshold value we used to screen differentially expressed lncRNAs and mRNAs was a fold change ≥2.0. Differences between two groups or more than two groups were evaluated for statistical significance by the independent *t*-test and One-Way ANOVA, respectively, using SPSS v21.0 software (SPSS Inc., Chicago, IL). Statistical significance was set at *p* < 0.05.

## 3. Results

### 3.1. The Differentially Expressed lncRNA/mRNA during the Osteogenic Differentiation of hBMMSCs

**Figure 1:**
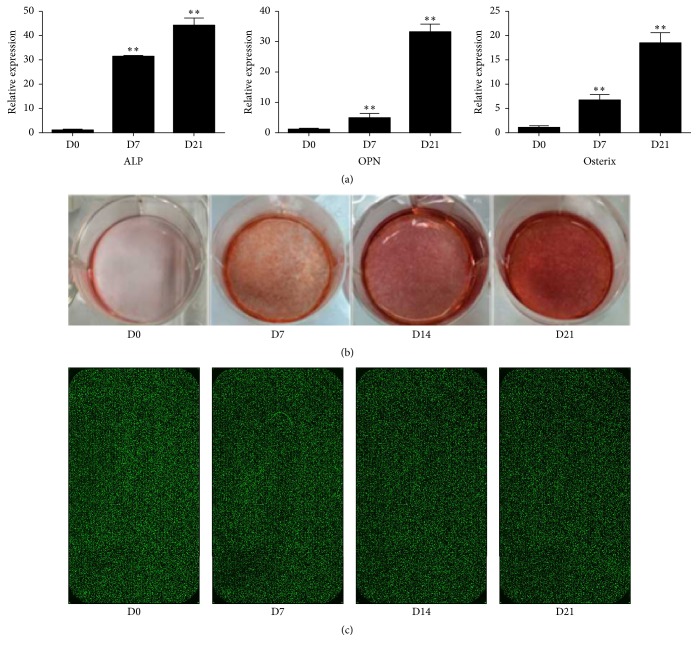
(a) The expression levels of osteogenic markers ALP, OPN, and Osterix were significantly increased following osteogenic induction (^*∗∗*^*p* < 0.01). (b) The staining intensity of Alizarin Red S was increased following osteogenic induction. (c) Microarray analysis of gene expression during osteogenic differentiation of hBMMSCs.

The expression levels of osteogenic markers including ALP, OPN, and Osterix as well as Alizarin Red S staining intensity were significantly increased after in vitro induction of osteogenic differentiation (Figures 1(a) and 1(b)).

**Figure 2:**
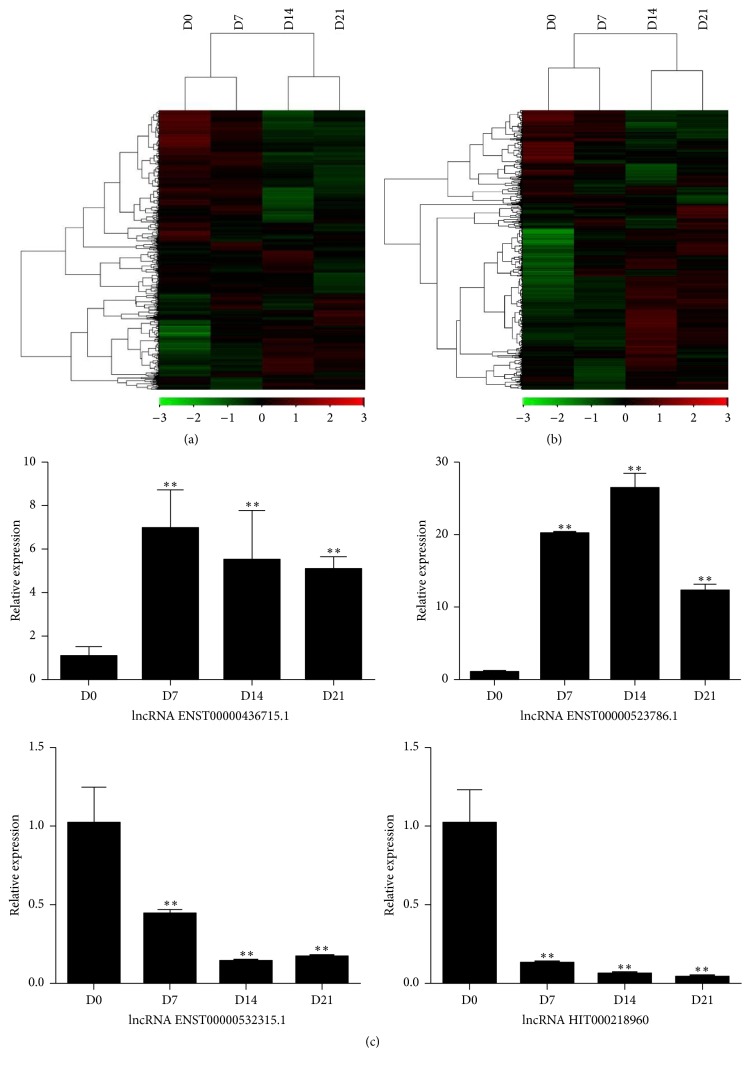
(a) The hierarchical cluster analysis revealed the differentially expressed lncRNAs during the osteogenic differentiation of hBMMSCs. (b) The hierarchical cluster analysis revealed the differentially expressed mRNAs during the osteogenic differentiation of hBMMSCs. (c) qPCR validation of the significantly different expressed lncRNAs during the osteogenic differentiation of hBMMSCs. ^*∗∗*^indicates *p* < 0.01.

The results of microarray assay were shown in Figure 1(c). The analysis revealed that 923 lncRNA, 1393 lncRNA, and 1338 lncRNA were significantly increased (>2 fold) in cells cultured in osteogenic medium at D7, D14, and D21, respectively, compared to those at D0, while 993 lncRNA (D7), 3843 lncRNA (D14), and 3688 lncRNA (D21) were remarkably decreased (≤2 fold). Similarly, compared with the controls (D0), 1462 mRNA (D7), 4093 mRNA (D14), and 3354 mRNA (D21) were significantly upregulated while 953 mRNA (D7), 2236 mRNA (D14), and 1923 mRNA (D21) were downregulated more than two times. The hierarchical cluster analysis revealed the differentially expressed lncRNAs (Figure 2(a)) and mRNAs (Figure 2(b)) during the osteogenic differentiation of hBMSCs. The red and the green shades indicated the expression above and below the relative expression. Among these significantly differentially expressed lncRNA/mRNA, 433 lncRNAs and 956 mRNAs were continuously upregulated. While 232 lncRNAs and 229 mRNAs were continuously downregulated. The representative consistent upregulated/downregulated lncRNAs and mRNAs were summarized in Tables 1–4.

Then we chose four consistently upregulated/downregulated lncRNAs for qRT-PCR validation. qRT-PCR results revealed the expression levels of ENST00000523786.1 and ENST00000436715.1 were significantly upregulated during hBMMSC osteogenic differentiation (*p* < 0.01), while ENST00000532315.1 and HIT000218960 expression were downregulated (*p* < 0.01) (Figure 2(c)). The qRT-PCR results further corroborated that our microarray data was convincing.

### 3.2. Bioinformatics Analysis of Differentially Expressed lncRNAs and mRNAs during Osteogenesis of hBMMSCs

**Figure 3:**
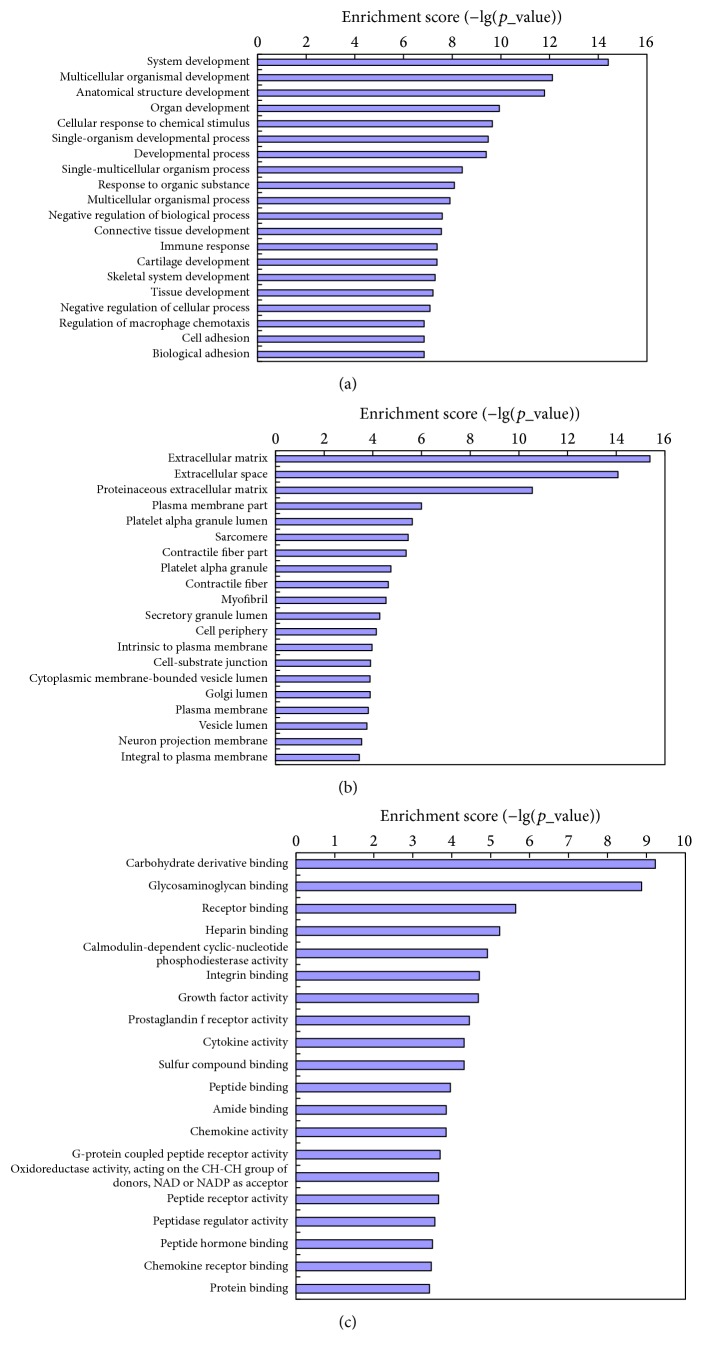
GO analysis of the significantly affected biological process, cellular component, and molecular function during osteogenic differentiation of hBMMSCs.

Six hundred and eighty significantly differentially expressed mRNAs were enriched in biological processes during osteogenic differentiation of hBMMSCs and system development hit the highest score. As regards cellular component, there were 702 differentially expressed mRNAs. Among them, the extracellular matrix (ECM) contained most abundant differentially expressed genes. Six hundred and thirty-one differentially expressed mRNAs were enriched in molecular function and carbohydrate derivative binding had the highest score (Figures 3(a)–3(c)).

**Figure 4:**
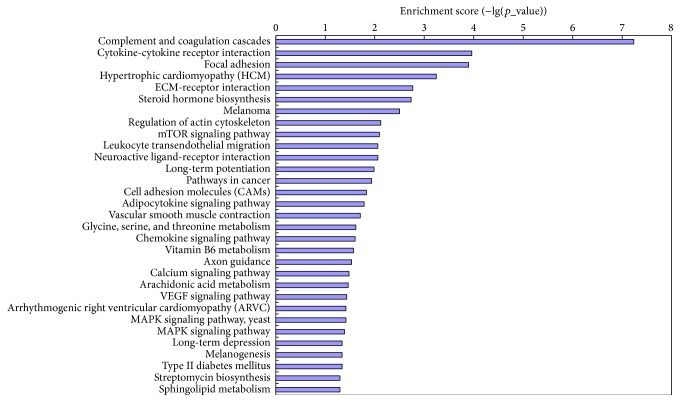
KEGG analysis of the significantly affected biological pathways during osteogenic differentiation of hBMMSCs.

KEGG analysis showed that there were 427 differentially expressed mRNAs enriched in 31 biological pathways during osteogenesis of hBMMSCs. The pathway with highest score was complement and coagulation cascades, which included 21 differentially expressed genes (Figure 4).

### 3.3. The Effects of lncRNA ENST00000502125.2 Upregulation/Downregulation on the Osteogenic Differentiation of hBMMSCs

**Figure 5:**
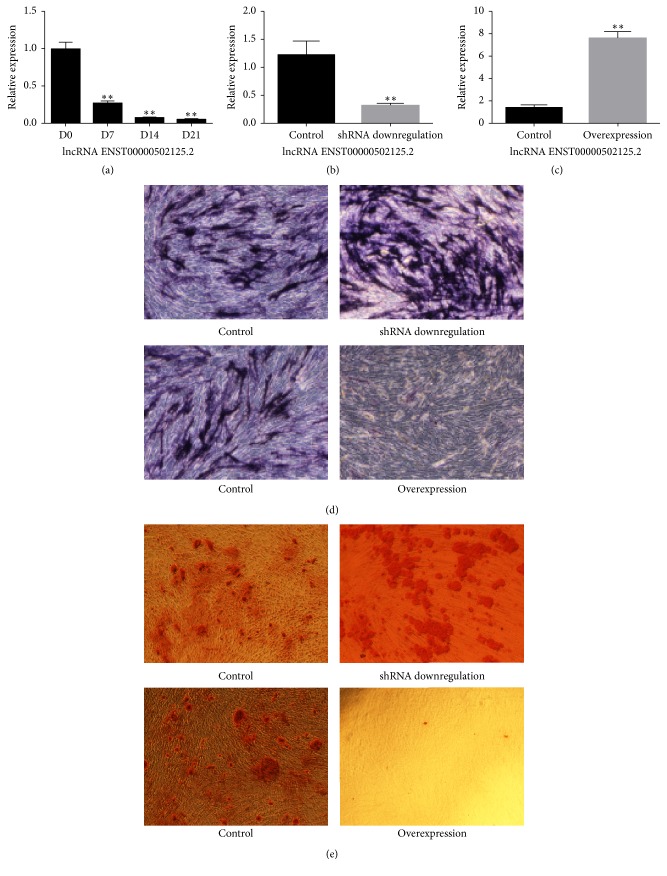
(a) The expression level of lncRNA ENST00000502125.2 was decreased during osteogenic differentiation of hBMmSCs. (b) ShRNA could significantly downregulate lncRNA ENST00000502125.2 expression. (c) Lentivirus vectors could significantly upregulate lncRNA ENST00000502125.2 expression. (d) Downregulation of lncRNA ENST00000502125.2 promoted the ALP staining intensity, and vice versa (200x). (e) Downregulation of lncRNA ENST00000502125.2 promoted Alizarin Red S staining intensity, and vice versa (200x). ^*∗∗*^indicates *p* < 0.01.

The expression level of lncRNA ENST00000502125.2 was significantly decreased during osteogenic differentiation of hBMMSCs (Figure 5(a)).

The target sequences we designed could upregulate or downregulate lncRNA ENST00000502125.2 with high efficiency (Figures 5(b) and 5(c)). Our results showed that the ALP staining intensity was significantly stronger in hBMMSCs subjected to lncRNA ENST00000502125.2 downregulation. In addition, the number of nodules was significantly more in the lncRNA ENST00000502125.2 knockdown cells. However, the staining intensities of ALP and Alizarin Red S assays were weaker when lncRNA ENST00000502125.2 was overexpressed (Figures 5(d) and 5(e)).

## 4. Discussion

LncRNAs had long been considered as nonfunctional junk [14]. However, recent studies showed that lncRNAs play a central role in regulating a number of biological processes including, but not limited to, transcription regulation, epigenetic modification, DNA methylation, and histone modification [15]. Deregulation of lncRNAs has been associated with the progression of many diseases such as cancer and cardiovascular diseases [16, 17].

Previous study have screened the expression profile of lncRNAs during osteogenic differentiation of MSCs [18]. However, the role of lncRNAs in osteogenesis remains poorly known. In this study, a number of novel lncRNAs and mRNAs especially those consistently deregulated might regulate hBMMSC osteogenic differentiation. For instance, both leukemia inhibitory factor (LIF) and frizzled-related protein (FRZB) are critical for osteogenesis [19, 20]. Real-time PCR was performed to validate our microarray findings. The reason we chose ENST00000523786.1, ENST00000436715.1, ENST00000532315.1, and HIT000218960 for microarray validation was that these molecules were consistently upregulated or downregulated during osteogenic differentiation of hBMMSCs. Most of these lncRNAs and mRNAs were first-time identified. GO analysis was performed to further annotate the biological function of differentially expressed mRNAs and a significant amount of GO terms were associated with developmental processes. Most enriched biological processes were correlated with osteogenic differentiation such as cartilage development, skeletal system development, and developmental process. For cellular component, ECM has been demonstrated to play an important role in regulating osteogenesis. For molecular function, integrin binding and growth factor activity are related with osteogenic differentiation. KEGG pathway analysis showed that many pathways such as cytokine-cytokine receptor interaction and ECM-receptor interaction were associated with osteogenic differentiation. To evaluate the genes regulated by lncRNAs, we selected two significantly deregulated lncRNAs (lncRNA ENST00000585537.1 and lncRNA eHIT000015952) for further analysis. In combination with the information of mRNAs, IncRNA ENST00000585537.1 was found to be located on chromosome 17 and three protein-coding genes (MAP2K6, TEKT1, and ABCA5) were near its 300 kb area. For lncRNA eHIT000015952, it was located on chromosome 3 and seven protein-coding genes (such as PAK2, MFI2, and DLG1) were near its 300 kb area (data not shown).

Through microarray analysis, we identified a number of lncRNAs potentially affecting osteogenic differentiation. LncRNA ENST00000502125.2 was chosen for further study as it was consistently downregulated during osteogenic differentiation of hBMMSCs, indicating that it might be a potent regulator of osteogenesis. LncRNA ENST00000502125.2 inhibition promoted the osteogenic differentiation of hBMMSCs and vice versa, suggesting it might play a critical role in osteogenesis. To the best of our knowledge, this was the first time to reveal this novel function of lncRNA ENST00000502125.2. Consistent with our findings, ectopic expression of lncRNA H19 promoted osteogenic differentiation of hMSCs both in vitro and in vivo, and opposite results were found when lncRNA H19 was inhibited. In addition, miR-675 was identified as a downstream target of lncRNA H19 [21]. Jin et al. reported that knockdown of lncRNA MIR31HG not only increased osteogenic differentiation of human adipose-derived stem cells both in vitro and in vivo, but also maintained this promotion effect even under the inflammatory environment, indicating targeting lncRNA MIR31HG might be an effective strategy to improve the osteogenic capacity of stem cells in bone engineering [13]. Similarly, lncRNA NONHSAT009968 suppression ameliorated staphylococcal protein A-inhibited osteogenic differentiation in hBMMSCs [22]. These studies demonstrate that lncRNAs are important regulators of osteogenic differentiation.

## 5. Conclusions

Collectively, our microarray data provides novel information regarding the potential role of mRNAs and lncRNAs in regulating the hBMMSC osteogenic differentiation. We also identify the top enriched GO and KEGG pathways. Moreover, lncRNA ENST00000502125.2 is demonstrated to be a negative regulator of osteogenic differentiation, indicating it might be a novel therapeutic target for promoting bone formation.

## Supplementary Material

Supplementary table 1 provides the primer sequences of mRNAs and IncRNAs for qRT-PCR.

## Figures and Tables

**Table 1:** The consistent upregulated lncRNAs in D7 group, D14 group, and D21 group compared to D0 group.

Probe name	Fold change			lncRNA ID	Chromosome	Strand
	D7 versus D0	D14 versus D0	D21 versus D0			
p4114	21.62478164	11.60060386	25.92556994	ENST00000418923.1	13	−
p38401_v4	30.86834436	39.89716139	47.83972529	ENST00000390168.4	11	−
p35386_v4	8.850828267	13.50510134	8.569022202	ENST00000524338.1	8	−
p33814	10.7500145	19.33207243	15.16926732	uc021wsx.1	3	−
p34143_v4	17.84687532	21.39256843	22.59689433	ENST00000428066.1	11	−
p20779	11.0580876	8.572849402	12.7458369	TCONS_00004149	2	−
p29982	14.36987842	18.31301009	19.1400538	TCONS_00015010	8	−
p34144_v4	9.448857515	11.1571628	12.01925275	ENST00000447298.1	11	−
p13783	29.95472326	22.41719711	21.2711142	ENST00000507217.1	5	+
p41550_v4	24.25949023	36.60054941	28.7447275	XR_242466.1	8	−
p15703	16.07037289	23.13735751	15.39810811	ENST00000521483.1	8	−
p35387_v4	13.86215164	20.48568771	12.3106551	ENST00000518943.1	8	−
p33593	8.765900177	18.72201809	18.08884202	ENST00000454645.1	9	−
p1239	8.87593437	29.58522619	15.2389718	ENST00000423943.1	1	+
p26385	8.374898467	9.089043638	11.37470244	uc003tcq.1	7	−
p23967	15.48067958	19.56789008	13.49101323	TCONS_00015014	8	−
p23964	10.21976098	12.64248095	12.07873525	TCONS_00015011	8	−
p15702	15.66396998	21.61260698	15.13024559	ENST00000523786.1	8	−
p23883	11.98005378	16.83343272	9.64895952	TCONS_00013634	7	+
p15704	11.4736364	19.16351897	11.40714697	ENST00000523664.1	8	−

**Table 2:** The larger continuous downregulated lncRNA in D7 group, D14 group, and D21 group compared to D0 group.

Probe name	Fold change			lncRNA ID	Chromosome	Strand
	D7 versus D0	D14 versus D0	D21 versus D0			
p26805	−6.180586296	−18.31712024	−21.76631127	eHIT000015952	3	−
p26850	−3.162097824	−7.896981442	−5.113394731	HIT000062015	5	+
p6751	−3.075056887	−9.43623037	−4.115732163	ENST00000567574.1	17	−
p11958	−3.095430977	−11.50752842	−4.973522833	ENST00000464242.1	3	+
p43709_v4	−3.119120027	−6.225875253	−6.275615097	NR_109889.1	20	−
p18116	−3.243406388	−5.670092378	−5.470304342	TCONS_00018160	10	+
p33827	−3.553411085	−4.48591536	−4.526856894	uc021zin.1	6	+
p41682_v4	−3.08170091	−3.169656935	−6.44910429	XR_242518.2	9	+
p24751	−3.332997418	−8.352157977	−4.184503053	XR_109875.3	1	−
p25066	−3.147224208	−7.23319858	−5.77391016	NR_026880.1	17	+
p7544	−3.526526302	−5.765265908	−7.295186192	ENST00000574526.1	17	+
p601	−3.396662015	−7.265009931	−6.836209784	ENST00000413035.1	1	−
p5379	−3.951455716	−6.197452204	−4.222822011	ENST00000561344.1	15	−
p5378	−3.263970006	−13.03398613	−8.189183014	ENST00000502125.2	15	−
p2545	−4.012409632	−8.23404424	−5.849793103	ENST00000532315.1	11	−
p26487	−3.084674582	−4.190874299	−4.371006429	uc004aaw.1	9	−
p34220_v4	−4.329673813	−3.902414327	−4.992448343	ENST00000564531.1	12	−
p7506	−3.071148345	−14.10807749	−5.324648534	ENST00000584660.1	17	+
p37929_v4	−3.347180055	−5.438810651	−5.171230814	ENST00000603042.1	6	−

**Table 3:** The consistent upregulated mRNA in D7 group, D14 group, and D21 group compared to D0 group.

Probe name	Fold change			mRNA ID
	D7 versus D0	D14 versus D0	D21 versus D0	
A_23_P157007	20.93078768	46.88202561	48.44960505	TMEM176B
A_23_P155786	40.92369481	33.41643908	40.18324588	SULT1E1
A_33_P3421923	45.38015715	161.8576338	207.750768	CADM3
A_23_P144549	49.02268505	21.60574241	73.13865038	IBSP
A_23_P41114	21.69170424	65.94353401	31.28494343	CSTA
A_23_P119562	23.82145451	36.79416271	34.50429618	CFD
A_24_P398147	36.09050187	42.0693231	50.64949923	NEBL
A_23_P94397	69.98586179	126.4123144	206.0673461	OMD
A_24_P208436	30.66909159	52.38832752	69.37444886	PDE1A
A_23_P363778	25.62987078	54.96002289	59.15291665	FRZB
A_23_P52761	80.20879313	39.88630678	30.92324555	MMP7
A_23_P82990	75.35249622	66.33124962	278.1804069	OGN
A_24_P48723	62.3488783	46.0152525	114.5446685	PTGIS
A_33_P3361636	65.19427159	149.0955822	296.7605893	MGP
A_23_P81158	43.72592716	109.8552875	125.7786547	ADH1C
A_33_P3252286	27.80470836	197.9730171	186.8343613	CRLF1
A_23_P307310	24.53975993	22.28423515	24.81066209	ACAN
A_24_P264943	154.5977058	192.3886928	423.5632762	COMP
A_24_P330263	43.2923737	32.38378473	35.02566896	EDNRB

**Table 4:** The consistent downregulated mRNA in D7 group, D14 group, and D21 group compared to D0 group.

Probe name	Fold change			mRNA ID
	D7 versus D0	D14 versus D0	D21 versus D0	
A_33_P3421178	−4.978203478	−6.689279838	−6.73293016	ENST00000552367
A_33_P3347241	−3.234257846	−8.689836302	−10.18643693	ENST00000420598
A_23_P430658	−4.081348909	−5.123030497	−4.953099079	HEYL
A_24_P122137	−9.239361785	−4.534924085	−5.997598307	LIF
A_23_P358709	−3.492627299	−6.212881338	−7.058185271	AHRR
A_33_P3240951	−6.366913923	−7.306438093	−6.10226131	DPF3
A_33_P3357658	−6.362807169	−9.205441781	−12.61825188	HMGA2
A_23_P19754	−4.978203478	−6.689279838	−6.73293016	CPA4
A_33_P3338693	−10.83785842	−9.210934927	−11.71527326	SNAP25
A_21_P0010759	−3.730747446	−9.78227973	−5.089176405	ENST00000458145
A_33_P3281667	−3.873687841	−5.554700388	−5.14477388	CNTNAP3B
A_24_P160401	−7.369096775	−4.833372717	−4.539895532	CDCP1
A_33_P3422728	−3.864556879	−6.005084014	−4.89254934	CNTNAP3
A_23_P106194	−5.131831246	−4.991628029	−4.329195734	FOS
A_33_P3325897	−3.057861576	−13.01790152	−4.632399252	MPL
A_24_P50368	−3.790519797	−12.29526394	−9.281170737	BLID
A_33_P3321372	−3.234257846	−8.689836302	−10.18643693	ENST00000377653
A_33_P3356392	−3.256778088	−5.440437604	−8.336994595	RGL3
A_33_P3265467	−5.491535497	−6.710400621	−4.29031983	ENST00000374375
A_33_P3263518	−4.855417658	−6.782055075	−5.473469073	ENST00000298953
