# Supplementary material for: The Critical Role of Long Noncoding RNA in Osteogenic Differentiation of Human Bone Marrow Mesenchymal Stem Cells
Source: Biomed Res Int. 2017 Apr 27;2017:5045827. doi: 10.1155/2017/5045827 (PMC5425846; doi:10.1155/2017/5045827)
Supplement: Supplementary file 1 — Supplementary table 1 provides the primer sequences of mRNAs and IncRNAs for qRT-PCR. [file 5045827.f1.doc]

**Supplementary table 1 The primer sequence for qRT-PCR**

| **Gene** | **Primer sequence** |
| --- | --- |
| h-Osterix F | 5’-CCACCTACCCATCTGACTTTG-3’ |
| h-Osterix R | 5’-CCACTATTTCCCACTGCCTT-3’ |
| h-ALP F | 5’-AGAATCTGGTGCAGGAATGG-3’ |
| h-ALP R | 5’-TCGTATTTCATGTCTCCAGGC-3’ |
| h-OPN F | 5’-AGGCTGATTCTGGAAGTTCTG-3’ |
| h-OPN R | 5’-CTTACTTGGAAGGGTCTGTGG-3’ |
| h-GAPDH F | 5’-TGTTGCCATCAATGACCCCTT-3’ |
| h-GAPDH R | 5’-CTCCACGACGTACTCAGCG-3’ |
| h-ENST00000523786.1 F | 5’-CTTGGAGAATCGGCTCCCAC-3’ |
| h-ENST00000523786.1 R | 5’-ACGAAAGGCTGGAAGTGTCAT-3’ |
| h-ENST00000436715.1 F | 5’-TTCCCCATCCACAGAAATGGT-3’ |
| h-ENST00000436715.1 R | 5’-ATGTTGGGCTGATGAGGTCTG-3’ |
| h-ENST00000532315.1 F | 5’-GGGTTGGAGAATGCCAGTCA-3’ |
| h-ENST00000532315.1 R | 5’-AGAGTTTGGGAGCAGAGGGA-3’ |
| h-HIT000218960 F | 5’-TGCCATTTAGCCATAACTACCCTT-3’ |
| h-HIT000218960 R | 5’-TTGCAGGCCAGAAATACATACG-3’ |
| h-ENST00000502125.2 F | 5’-TCATGCTCTCCCCCAACCTA-3’ |
| h-ENST00000502125.2 R | 5’-ACGCTTTCTGAGGCTTGAGT-3’ |
